# Oxidative Potential of Atmospheric Particulate Matter: A Review of the Role of Metal−Organic Interactions, Mechanistic Insights, and Key Determinants

**DOI:** 10.1021/acs.est.5c08535

**Published:** 2026-01-06

**Authors:** Manfei Lin, Jian Zhen Yu

**Affiliations:** 1 Jiangsu Provincial Key Laboratory of Environmental Engineering, 563606 Jiangsu Provincial Academy of Environmental Science, Nanjing 210036, China; 2 Department of Chemistry and Division of Environment, Hong Kong University of Science & Technology, Clear Water Bay, Kowloon 999077, Hong Kong, China; 3 Division of Environment, Hong Kong University of Science & Technology, Clear Water Bay, Kowloon 999077, Hong Kong,China

**Keywords:** oxidative potential, particulate matter, reactive oxygen species, acellular assays, metal−organic interactions, health effects of particulate matter

## Abstract

Oxidative stress, resulting from antioxidant depletion or excessive reactive oxygen species (ROS) production, is a key mechanism linking ambient particulate matter (PM) exposure to adverse health effects. The oxidative potential (OP) of PM, a measure of inhaled PM’s capacity to deplete antioxidants or generate ROS, is largely driven by transition metals (TMs) such as iron and copper. However, coexisting organic matter also modulates OP, both directly through its own redox activity and indirectly via interactions with TMs that alter redox cycling. This review synthesizes current understanding of TM−organic interactions and their influence on the OP of PM, as assessed by acellular assays. We discuss mechanistic insights, key determinants, and the complexity of these interactions. The importance of considering TM−organic interactions in evaluating aggregate OP from individual components and apportioning OP to specific chemical species is highlighted, with implications for mechanistic studies and health risk assessment.

## Graphical abstract

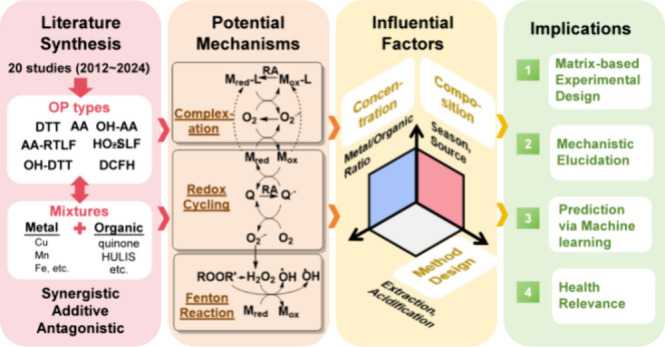

## 1 Introduction

Exposure to ambient particulate matter (PM) is associated with increased morbidity and mortality worldwide.^1^ A key mechanism underlying PM-induced health effects is oxidative stress, driven by depletion of endogenous antioxidants (e.g., ascorbic acid (AA), glutathione (GSH)) and overproduction of reactive oxygen species (ROS) in human or animal cells.^2–5^ This disruption of redox homeostasis is recognized as a central pathway linking PM exposure to adverse health outcomes.^6^ ROS comprise a family of oxygen-containing reactive molecules, including oxygen-centered radicals (e.g., superoxide radical (O_2_•^−^), hydroxyl radical (•OH)), some nonradical derivatives of oxygen (e.g., hydrogen peroxide (H_2_O_2_), singlet oxygen (^1^O), and hypochlorous acid (HClO)). Among these, •OH is particularly damaging due to its high reactivity with biomolecules including DNA, proteins, and lipids.^7^

The oxidative potential (OP) of PM, defined as its capacity to deplete antioxidants (i.e., reductants such as AA) or generate ROS, serves as a widely used proxy for its ability to induce oxidative stress. OP is commonly assessed using a range of approaches, including in vivo animal studies, in vitro cellular assays, and acellular chemical assays. While biological assays provide direct evidence of toxicological effects, they are labor-intensive, time-consuming, and require specialized equipment.^6,8^ Acellular assays, in contrast, offer rapid, cost-effective screening of OP, facilitating their use in epidemiological and toxicological research.^9,10^ Here, we designate OP end points by the target antioxidant or ROS measured (e.g., OP_DTT_ for dithiothreitol (DTT) consumption, OP_AA_ for AA depletion, OP_OH_ for •OH generation).

A variety of PM components contribute to OP, notably transition metals (TMs), quinones, and humic-like substances (HULIS).^9,11,12^ Among these, TMs such as iron (Fe), copper (Cu) and manganese (Mn) are major drivers of OP as measured by common assays (e.g., OP_DTT_, OP_AA_, OP_OH_).^13–15^ For instance, chelation of TMs with diethylene triamine pentaacetic acid (DTPA), a strong metal chelator, can suppress OP_DTT_ by ∼ 90% in PM extracts,^12^ and similarly inhibit OP_AA_ and OP_OH_ by 90−100%.^16^ Coexisting organic matter (OM) in PM, comprising a range of aromatic and aliphatic compounds with functional groups such as hydroxyl, carboxyl, and carbonyl moieties,^17,18^ can interact with TMs through chelation and redox cycling. Reduced organic nitrogen compounds, such as alkaloids, are also noteworthy OM constituents that possess functional groups capable of chelating TMs and thus possibly influence the OP.

Recent studies have increasingly recognized the importance and complexity of TM−organic interactions in determining OP of PM.^19–21^ While over 15 review articles published in the past decade have summarized advances in OP methodology,^22–24^ effects of PM characteristics (e.g., composition, sources, particle size, and aging),^9,10,25–30^ links to health outcomes,^9,10,22,30,31^ and methodological challenges,^22,26^ none have specifically focused on the impacts of TM−organic interactions on OP. Although some reviews briefly mention metal−organic complexes,^9,24^ a comprehensive synthesis of up-to-date findings and mechanistic insights is lacking.

In this review, we synthesize evidence from recent studies on the effects of TM−organic interactions on OP of PM, describing both consistencies and discrepancies observed across different OP assays, TMs, and organic species. We further summarize mechanistic understandings and key factors influencing these interactions. The objective of this review is to highlight the importance and complexity of TM−organic interactions in influencing the OP of inhaled PM.

## 2 Interaction Factors in Metal−Organic Mixtures

The interaction factor (*IF*), also referred to as the mixture effect index (*I*_*ME*_), is commonly used to quantify synergistic, antagonistic, or additive interactions in metal−organic mixtures.^19,21,32^ *IF* is defined as the ratio of the observed OP of the mixture over the arithmetic sum of the OPs of the individual components, as shown in eq 1:

$$\mathrm{IF}=\frac{\mathrm{OP}(\mathrm{mixture})}{\sum_{i}{\mathrm{OP}}_{i}}$$ 1

Where *OP*_*i*_ is the OP attributable to component *i*. An *IF* of 1 indicates an additive relationship among components, i.e., no mixture effect. *IF* values higher than 1 signify synergistic effects, while values less than 1 indicate antagonistic (suppressive) effects.

Table 1 and Figure 1 compile the *IF* values reported in 20 published studies (see Figure 1 caption), categorized by OP assay types and mixture compositions. Eight OP assays were included (OP_DTT_, OP_AA_, OP_AA-RTLF_, OP_OH-DTT_, OP_OH-AA_, OP_OH-SLF_, OP_H2O2-SLF_, and OP_DCFH_) and their definitions can be found in Table 1. Seven categories of mixtures were examined: metal mixtures, quinone mixtures, and five metal−organic mixtures (including quinones, carboxylates, imidazoles, secondary organic aerosol (SOA), and HULIS). Note that the first two categories, metal mixtures and quinone mixtures, are not metal−organic mixtures but are included here for their relevance in elucidating interaction mechanisms. The specific metal species, quinones, and other organic constituents investigated are detailed in Table 2. *IF* values were either directly extracted from the original publications or calculated from digitized data using Origin software.

Among the various OP assays, *IF*s in OP_DTT_ have been the most extensively investigated, followed by those in OP_AA_, with studies covering all seven types of mixtures summarized in Table 1. Among metal−organic mixtures, metal−quinone combinations have received the most attention, while metal−HULIS mixtures have also been the subject of several studies. *IF* values for these mixtures display a wide range; for example, *IF*s for OP_DTT_ in metal−quinone mixtures vary from 0.43 ± 0.01 to 2.69 ± 0.24 (Figure 1). To facilitate comparison, *IF*s for binary mixtures of specific species are shown in Figure 2. In comparison, studies on metal−carboxylate, metal−imidazole, and metal−SOA mixtures are limited. Despite some variability across studies, general trends in *IF* values are observed for different metal−organic mixtures. These trends are summarized below, while potential mechanisms and influencing factors are discussed in Sections 3 and 4.

**Table 1:** Definition of 8 Types of OP and Summary of IFs in 7 Types of Mixtures in Previous Studies

	OP	OP_DTT_	OP_AA_	OP_AA-RTLF_	OP_OH-DTT_	OP_OH-AA_	OP_OH-SLF_	OP_H2O2-SLF_	OP_DCFH_
	definition	DTT loss rate	AA loss rate	AA loss rate in synthetic respiratory tract lining fluid (RTLF)	•OH generation rate in the DTT assay	•OH generation rate in the presence of AA	•OH generation in surrogate lung fluid (SLF)	H_2_O_2_ generation in SLF	ROS by 2,7-dichlorofluoroscein (DCFH) assay
number of studies		11	5	1	3	3	3	2	2
IFs	metal + metal	0.33−1.88 ± 0.68	0.62 ± 0.04−1.02 ± 0.02	0.68−1.63	0 ± 0−1 ± 0		1.6	0.19	
	quinone + quinome	0.88 ± 0.12−1.00 ± 0.08	0.52 ± 0.17−2.64 ± 0.35	0.33−1.42	0.42 ± 0.07−2.03 ± 0.64				
	metal + quinone	0.43 ± 0.01−2.69 ± 0.24	1.02 ± 0.12−1.35 ± 0.10		0 ± 0−3.3 ± 0.6		0.83−2.02	0.2−1.07	
	metal + carboxylate	1.04 ± 0.22−2.78 ± 0.70	1.33−6.10			1.3−17.4			
	metal + imidazoles	0.37−1.37	0.29 ± 0.04−0.81 ± 0.08		0−2.21	0.24−1.35 ± 0.32			
	metal + SOA	0.50−0.87	0.75−2.93			1.24−1.99			0.27−1.16
	metal + HULIS	0.64 ± 0.18−1.88 ± 0.07	0.30 ± 0.11−1.69 ± 0.32		0.19−1.77	0.57 ± 0.15−2.08 ± 0.38	1.08−4.35 ± 0.35	0.95 ± 0.08−1.53 ± 0.28	0.97 ± 0.12−2.91 ± 0.42

**Figure 1:**
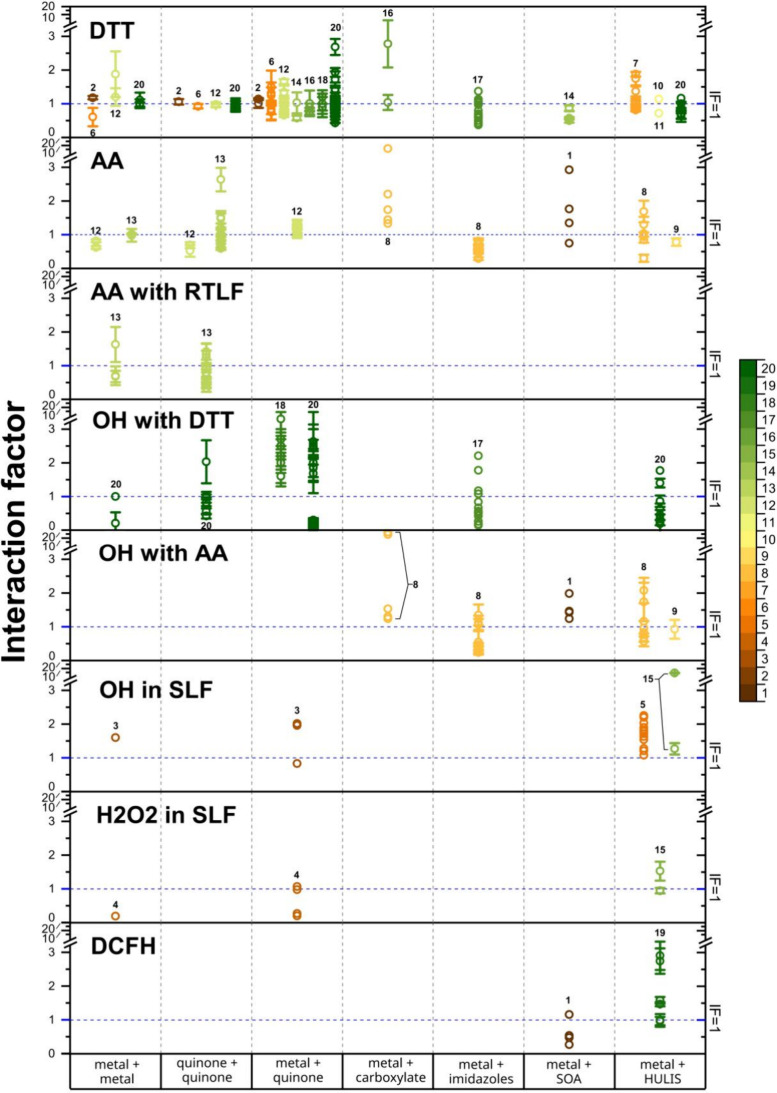
Summary of interaction factors (IF) categorized by types of OP and mixture composition, based on literature data. Symbols denote individual IF values; error bars indicate the corresponding standard deviations in the respective studies. The color scale (right, range: 1−20) identifies literature sources, with each color denoting one of the 20 studies listed below: 1, Campbell et al. (ref 33); 2, Charrier and Anastasio (ref 11); 3, Charrier and Anastasio (^34^); 4, Charrier et al. (ref 35); 5, Gonzalez et al. (ref 36); 6, Guo et al. (ref 19); 7, Lin and Yu (ref 37); 8, Lin and Yu­(ref 20); 9, Lin and Yu (ref 38); 10, Lin et al. (ref 39); 11, Lu et al. (ref 40); 12, Pietrogrande et al. (ref 41); 13, Souza et al. (ref 42); 14, Wang et al. (ref 43); 15, Wei et al. (ref 32); 16, Wu et al. (ref 44); 17, Wu et al. (ref 45); 18, Xiong et al. (ref 46); 19, Yan et al. (ref 47); and 20, Yu et al. (ref 21).

**Table 2:** Summary of Metals, Quinones, and Other Organic Compounds Evaluated in Mixture-Effect Studies

		reference^a^																			
category	species	1	2	3	4	5	6	7	8	9	10	11	12	13	14	15	16	17	18	19	20
metal	Fe	√	√	√	√	√	√		√			√	√	√		√	√		√	√	√
	Cu	√	√	√	√		√	√	√				√	√	√	√	√	√		√	√
	Mn						√	√						√						√	√
	hydrophilic fraction									√	√										√
quinone	9,10-phenanthrenequinone(PQ)		√	√	√		√						√	√			√		√		√
	1,2-naphthoquinone (1,2-NQ)		√	√	√		√						√	√	√				√		√
	1,4-naphthoquinone (1,4-NQ)						√							√	√		√		√		√
	5-hydroxyl-1,4-naphthoquinone (5-*H*-1,4-NQ)																		√		√
	1,4-benzoquinone (1,4-BQ)													√							
carboxylate	citrate								√								√				
	malonate								√												
	oxalate								√												
imidazoles	imidazole								√									√			
	2-methylimidazole								√									√			
	2,4-dimethylimidazole								√									√			
	2-ethyl-4-methyl imidazole								√												
	pyridine																	√			
SOA	naphthalene SOA	√													√						
	phenanthrene SOA														√						
	α-pinene SOA														√						
	β-pinene SOA	√																			
	limonene SOA														√						
HULIS	SRFA					√										√				√	√
	SRHA																			√	
	HULIS							√	√	√	√	√									√

a Refer to Figure 1 caption for the numbered references. Each tick indicates that the corresponding species was examined in that study.

### Metal-Quinone Mixtures

*IF* values vary substantially depending on both the metal and the OP assay (Figure 2). For OP_AA_, median *IFs* were 1.21 for Cu (range: 1.02 ± 0.12 to 1.35 ± 0.10) and 1.12 for Fe (range: 1.05 ± 0.09 to 1.24 ± 0.08), indicating slight synergistic effects.^41^ For OP_DTT_, interaction effects were both metal- and quinone-specific: Cu generally exhibited antagonistic effects (median: 0.80, IQR: 0.68 ± 0.05 to 0.97 ± 0.04), Fe showed largely additive effects (median: 1.00, IQR: 0.9 ± 0.1 to 1.05 ± 0.05), and Mn demonstrated synergistic effects (median: 1.32, IQR: 0.95 ± 0.05 to 1.50 ± 0.50) across seven studies.^11,19,21,41,43,44,46^ Quinone identity also played a role: 9,10-phenanthrenequinone (PQ) showed stronger suppression on OP_DTT_ by Cu, while 1,4-naphthoquinone (1,4-NQ) led to larger enhancement of OP_DTT_ by Mn. For OP_OH-DTT_, *IF*s revealed different metal-specific effects compared to OP_DTT_, with significant synergistic effects for Fe (median: 2.34, IQR: 1.87 ± 0.29 to 2.52 ± 0.17) and pronounced antagonistic effects for both Cu and Mn.^21,46^

**Figure 2:**
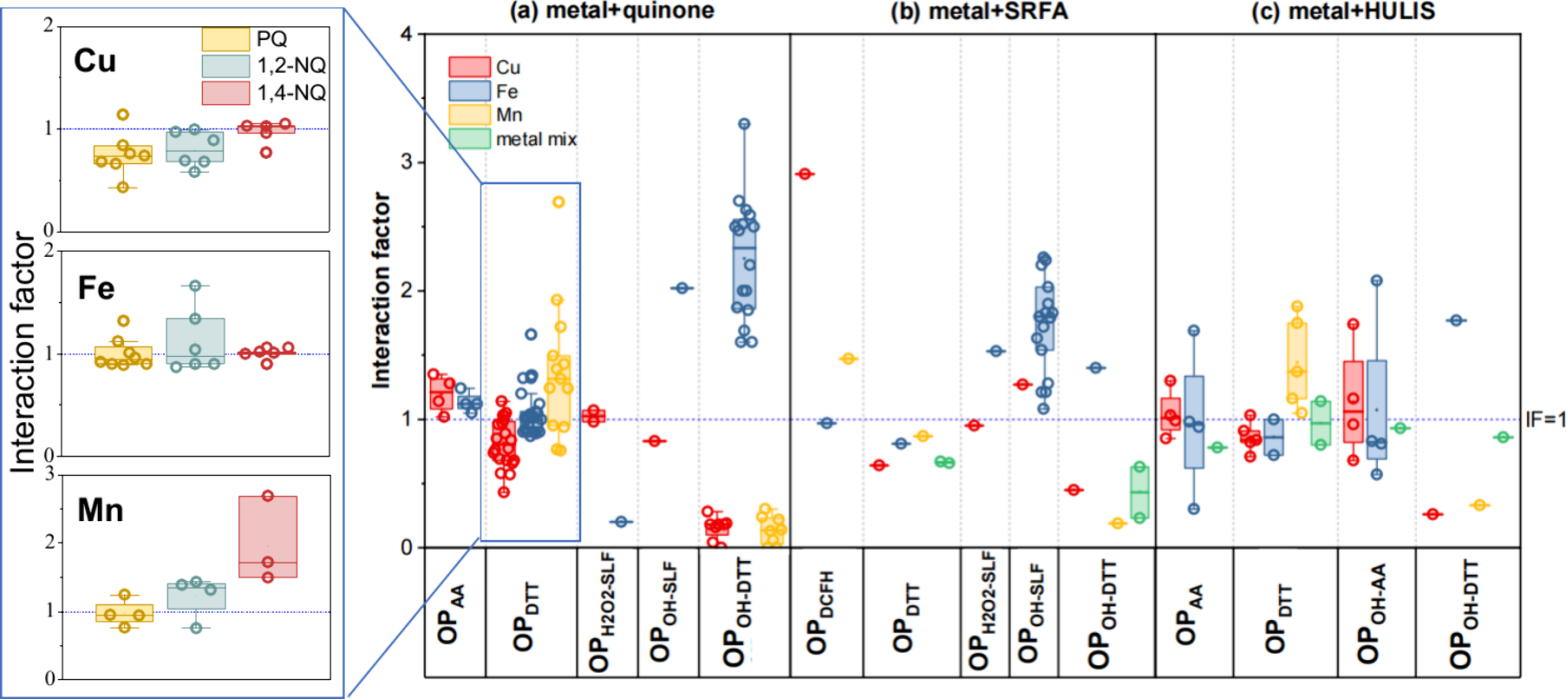
Interaction factor (IF) values for three mixture systems: (a) metal-quinone, (b) metal-SRFA, and (c) metal-HULIS. Left panel: colors represent quinone types (PQ, 1,2-NQ, and 1,4-NQ). Right panel: colors denote metal types (Cu, Fe, Mn, or metal mixture). The dashed line at IF = 1 indicates an additive (no-interaction) effect.

### Metal-HULIS Mixtures

Recent studies have used Suwannee River fulvic acid (SRFA) as a surrogate for HULIS.^21,32,36,47^ Although data are limited (except for OP_OH-SLF_ of Fe−SRFA), *IF* values for metal−SRFA mixtures generally mirror those for metal−quinone mixtures. For example, Cu−SRFA mixtures show more pronounced antagonistic effects for OP_DTT_ than Fe or Mn. Fe−SRFA mixtures exhibit significant synergistic effects for OP_OH-SLF_ (median: 1.72, IQR: 1.28 to 1.9), similar to Fe−quinone mixtures. Similarly, for OP_OH-DTT_, antagonistic effects are observed for Cu and Mn, while Fe exhibits synergism in metal−SRFA mixtures. Overall, interaction effects in metal−HULIS mixtures are consistent with those seen in metal−quinone systems, particularly for OP_DTT_ and OP_OH-DTT_.^21,37,39,40^

### Metal-Carboxylate Mixtures

Synergistic effects have been reported for OP_DTT_,^44^ as well as OP_AA_ and OP_OH-AA_.^20^

### Metal-Imidazole Mixture

Antagonistic effects are consistently observed, with median *IF*s of 0.64 for OP_DTT_ and 0.50 for OP_OH-DTT_ in the DTT assay,^45^ and 0.67 for OP_AA_ and 0.46 for OP_OH-AA_ in the AA assay.^20^

### Metal-SOA Mixtures

Interaction effects differ by assay and SOA type. Antagonistic effects are observed for OP_DTT_ in mixtures of Cu and PAH-derived SOA, while no significant interaction is seen for Cu and α-pinene SOA.^43^ In contrast, synergistic effects are found for OP_AA_ and OP_OH-AA_ in mixtures of Fe/Cu and naphthalene/β-pinene SOA.^33^

## 3 Current Understandings of IFs

This section summarizes current mechanistic insights into interaction effects observed in two commonly adopted assays: the DTT assay and the AA assay, corresponding to OP_DTT_, OP_OH-DTT_, OP_AA_, and OP_OH-AA_. We focused on DTT and AA assays primarily for two reasons. First, these assays have been extensively studied in the literature, enabling relatively robust mechanistic interpretations. Second, compared to more complex systems (e.g., SLF with multiple antioxidants/ligands), their simpler compositions minimize interference, facilitating clearer elucidations of the specific interactions between PM chemical species and OP. Our focus is on *IF*s arising specifically from interactions between TMs and organics. Interaction effects unrelated to direct TM−organic interplay—such as the antagonistic OP_OH-DTT_ observed for Cu or Mn mixtures, which is attributed to the absence of •OH formation in their reaction mechanisms^48^—are beyond the scope of this review.

Understanding *IFs* requires elucidating how TMs and organics interact. Figure 3 schematically summarizes the main mechanisms by which TM−organic interactions influence OP: metal−organic complexation, redox cycling, and Fenton­(-like) reactions. In the absence of strong reducing agents, redox-active metals like Cu­(II) and Fe­(III) can be reduced by hydroquinones (QH_2_) or semiquinone radicals (Q•−), thereby accelerating quinone redox cycling.^49,50^ However, when a strong reducing agent is present, these redox reactions between quinones and TMs become negligible. Accordingly, this review does not consider TM−organic redox cycling under such conditions.

**Figure 3:**
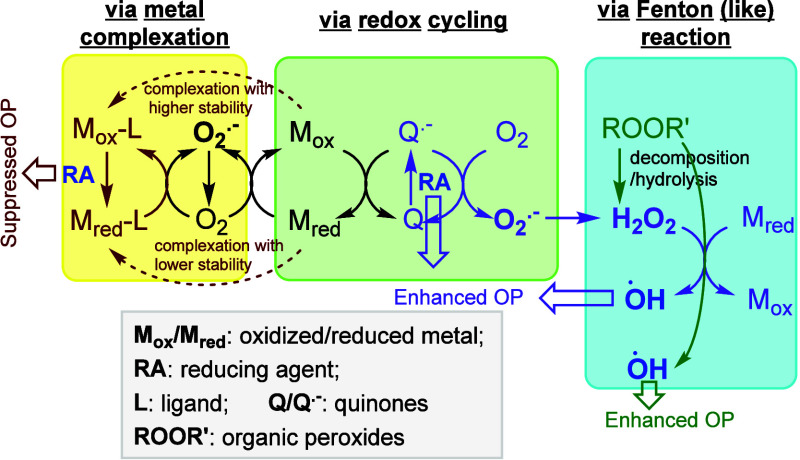
Schematic diagram of TM−organics interactions and their influences on OP.

### 3.1 Impacts on OP through Metal−Organic Complexation

It is well established that organic compounds in PM_2.5_ can form complexes with TMs. Direct evidence for such complexation can be obtained via multiple analytical approaches, as detailed below. For instance, specific metal−organic complexes (e.g., C_12_H_6_N_2_O_8_Fe^−^ and C_6_H_3_NO_4_FeCl_2_^−^) were identified using Quadrupole/time-of-flight mass spectrometry.^44,51^ The ^1^H-nuclear magnetic resonance (^1^H NMR) and Fourier-transform infrared (FTIR) spectra of HULIS and HULIS-Cu­(II) revealed that Cu­(II) mainly bound with aromatic HULIS.^52^ Additionally, TMs (e.g., Cu­(II), Mn­(II)) showed quenching effects on the fluorescence of HULIS or water-soluble organic carbon (WSOC) in PM_2.5_, indicating TM-organic interactions, as evidenced by the excitation−emission matrix fluorescence spectroscopy.^52,53^ For clarity, we classify these ligands into two categories based on their redox activity: “nonredox” ligands (e.g., carboxylic acids,^54^ imidazoles^55^) and “redox” ligands (e.g., quinones, especially those with ortho-positioned oxygen atoms^43^).

#### 3.1.1 Impacts of “Nonredox” Ligands

The formation of metal−ligand complexes can alter the reduction potential of TMs, as described by the Nernst equation:

$$E_{1}=E_{0}-\frac{RT}{nF}\ln\frac{\beta_{ox}}{\beta_{red}}$$ 2

where *E*_1_ and *E*_0_ are the reduction potentials of the metal complex and uncomplexed metal, respectively, and β_*ox*_ and β_*red*_ are the overall stability constants for the oxidized and reduced forms of the metal complex, respectively. Generally, “hard” ligands (e.g., carboxylates) preferentially bind “hard” metals, while “soft” ligands (e.g., imidazoles) bind “soft” metals,^56^ in which “hard” refers to highly charged and lowly polarized. For carboxylate ligands, $\ln\frac{\beta_{ox}}{\beta_{red}}$ is positive, lowering the reduction potential and favoring TM oxidation. In contrast, reduced nitrogen ligands (e.g., imidazoles) increase the reduction potential, suppressing TM oxidation. Because antioxidant consumption and •OH formation rely on TM redox cycling, the nature of the ligand directly affects measured OP values. This mechanism explains observed synergistic effects in TM−carboxylate mixtures and antagonistic effects in TM−imidazole mixtures on OP_AA_, OP_OH-AA_,^20^ as well as OP_DTT_ and OP_OH-DTT_.^45^ The magnitude of suppression by imidazoles correlates with their binding affinity to Cu (imidazole ≈ 2-methylimidazole >2,4-dimethylimidazole), suggesting a mechanistic link between complexation strength and OP suppression.^20^

#### 3.1.2 Impacts of “Redox” Ligands on OP_DTT_

The *IF*s for metal−quinone mixtures have been most frequently studied using the DTT assay (Figure 1). *IF*s are both metal- and quinone-specific: Cu−quinone mixtures typically display antagonistic to additive effects, while Mn−quinone mixtures range from additive to synergistic, following the sequence PQ < 1,2-NQ < 1,4-NQ (Figure 2).

In principle, ortho-quinones (e.g., PQ, 1,2-NQ) have higher electron density on oxygen atoms and greater electron delocalization (PQ > 1,2-NQ > 1,4-NQ), which confers stronger binding affinities to metals such as Cu or Mn. Consistent with this, antagonistic effects are strongest for Cu−PQ, moderate for Cu−1,2-NQ, and negligible for Cu−1,4-NQ mixtures (Figure 2).^21,41,43^ ^1^H NMR studies confirm the coordination of adjacent oxygen atoms in 1,2-NQ with Cu,^43^ supporting the role of binding affinity in modulating OP.

The mechanisms underlying the suppression of OP_DTT_ by Cu−quinone complexation have not been explicitly addressed in previous studies. Here, we propose hypotheses considering how complexation alters the reactivity of both transition metals and quinones (Figure 4). For Cu-catalyzed DTT oxidation, the mechanism described by Kachur et al.^48^ is widely accepted. Rather than free Cu ions, the Cu−DTT complex serves as the catalytically active species. Cu­(II), with its four-coordination property, can chelate two DTT molecules, facilitating intramolecular electron transfer from DTT sulfur to Cu­(II), followed by O_2_ reduction to an oxygen-containing intermediate. Quinones can also catalyze DTT oxidation by O_2_. Ortho-quinones, in particular, are more efficient,^57,58^ likely due to higher localized electron density on oxygen, which facilitates electron transfer from DTT sulfur to quinone oxygen via the π-conjugated system. Notably, previous studies observed PQ concentrations decline only slightly after reactions with DTT, while 1,2-NQ and 1,4-NQ are more substantially consumed, implying differences in their mechanisms − 1,2-NQ and 1,4-NQ undergo both thiol arylation and redox cycling.^57,59^ In Cu−quinone mixtures, the observed antagonistic effect on OP_DTT_ likely arises from two factors: (1) competitive binding, which disrupts the formation of the Cu−DTT complex and thus its catalytic activity, and (2) reduced electron density on quinone oxygen atoms, which diminishes electron transfer from DTT sulfur to quinone oxygen. Both mechanisms result from Cu−quinone complexation and lead to suppressed DTT oxidation.

**Figure 4:**
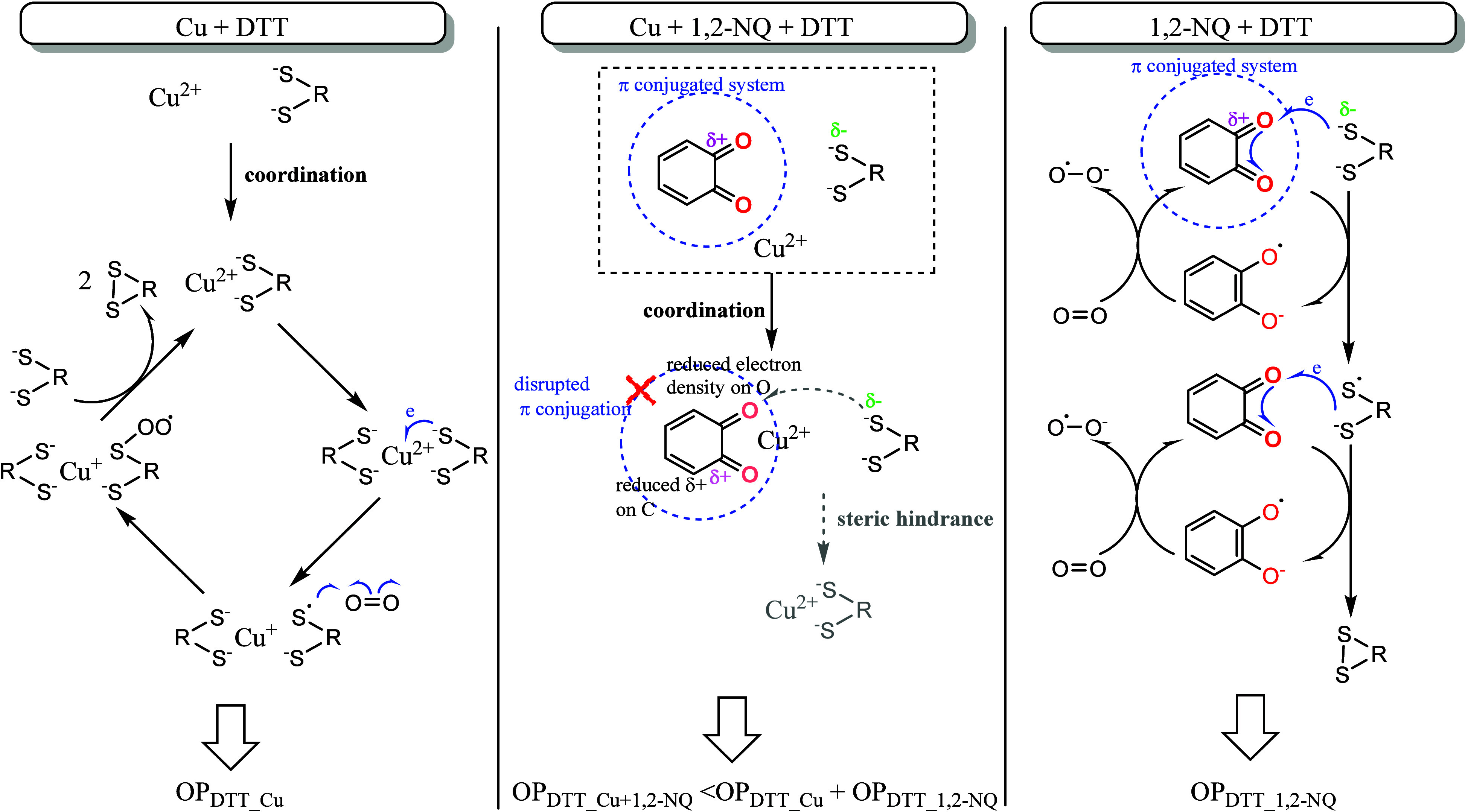
Schematic representation of the proposed mechanism for OP_DTT_ suppression resulting from Cu-1,2-NQ complexation. The Cu-DTT mechanism (left panel) is based on Kachur et al.^48^ while the 1,2-NQ-DTT mechanism (right panel) is based on Motoyama et al.^57^

A similar rationale applies to Mn-catalyzed DTT oxidation, given the lack of •OH production observed for both Cu and Mn in the DTT assay.^21^ Our previous work suggests that Mn­(III) is the active oxidant for DTT, but it is unstable and tends to disproportionate to MnO_2_ and Mn­(II).^37^ The competition between DTT oxidation by Mn­(III) and spontaneous Mn­(III) disproportionation is of importance. Chelation with certain organics (e.g., citrate, malonate, lactate) can stabilize Mn­(III).^60–62^ Thus, Mn−quinone complexation may stabilize Mn­(III), suppress disproportionation, and enhance DTT oxidation by Mn. The net effect in Mn−quinone mixtures is likely determined by the balance between synergistic and antagonistic interactions. Take the data from Yu et al.^21^ as an example (0.25 μM quinone and 0.1 μM metal). The contribution of quinone to total OP_DTT_ decreased from ∼ 90% in Mn−PQ mixtures to ∼ 60% in Mn−1,4-NQ mixtures. This suggests that as the influence of quinone reactivity modification via complexation diminishes, the effect of altered metal reactivity becomes more prominent. Consequently, *IF* values increase from Mn−PQ to Mn−1,4-NQ mixtures, consistent with the observations.

### 3.2 Impacts on OP through Fenton (Like) Reactions

The Fenton (like) reaction is a widely accepted mechanism underlying the synergistic effects observed in OP_OH-DTT_ of Fe-organic mixtures,^21^ OP_AA_,^33,41^ and OP_OH-AA_.^33^

#### 3.2.1 Enhanced OP in the Presence of Metal and Quinones

Quinones can oxidize DTT and AA, generating O_2_•^−^ and subsequently H_2_O_2_.^46,63^ TMs such as Fe, Cu and Mn are capable of reacting with H_2_O_2_ via Fenton (like) reaction.^64^ In contrast to Fe which exhibits optimal activity under acidic conditions, Cu-based and Mn-based Fenton systems can operate efficiently in neutral solutions. However, Cu­(I) is prone to oxidation by O_2_ to Cu­(II), reducing the availability of Cu­(I) for reaction with H_2_O_2_. As a result, Cu-based Fenton systems require a greater stoichiometric excess of H_2_O_2_ relative to Fe-based systems, so as to compensate for the competitive scavenging by O_2_.^64^ It is generally hypothesized that the synergistic effects on OP related to •OH production result from the Fenton reaction, where H_2_O_2_ generated by quinone-catalyzed DTT (or AA) oxidation is efficiently converted to •OH in the presence of TMs.^21,46^ Furthermore, AA is relatively unreactive toward peroxides and even less sensitive to larger organic peroxides with increased steric hindrance.^33^ It is hypothesized that enhanced •OH generation also leads to greater AA depletion, increasing OP_AA_.^33^

However, Miller et al.^65^ found that inorganic Fe­(II) reacts with H_2_O_2_ at pH 8.2 to produce a species other than •OH, in contrast to the well-known formation of •OH at low pH. Similarly, Wei et al.^32^ showed that Fe alone produced negligible •OH in the presence of H_2_O_2_ at neutral pH, whereas Fe−HULIS mixtures strongly promoted •OH formation. Kinetic modeling studies further indicated that Fe binding by SRFA ligands accelerates both O_2_•^−^ formation and H_2_O_2_ decomposition relative to Fe alone.^36^ Notably, the synergy observed in Cu−naphthalene SOA mixtures (which contain quinones) is not captured by kinetic models unless metal−organic complexation is considered.^33^ These findings highlight that metal complexation is essential for efficient H_2_O_2_ conversion to •OH under physiological conditions, underscoring its role in enhancing OP.

#### 3.2.2 Impact of Organic Peroxides in SOA

Organic peroxides contribute significantly to biogenic SOA, comprising 40−100% of α-pinene SOA.^43^ Decomposition or hydrolysis of hydroxyhydroperoxides, peroxy acids, and related organic peroxides generates H_2_O_2_,^66^ which can further participate in Fenton (like) reactions with TMs to enhance OP. Decomposition of ROOH (ROOH → RO• + •OH) is also promoted in the presence of Fe, primarily via Fenton-like heterolytic cleavage of the O−O bond.^67^ Tong et al. also demonstrated that •OH formation efficiency in Fe­(II)−SOA aqueous mixtures mirrors the order of the relative content of ROOH in SOA derived from α-pinene, β-pinene, and limonene.^67^ These results indicate that organics with peroxide structures can enhance OP by promoting Fenton (like) reactions and subsequent •OH formation.

## 4 Influential Factors on IF Values

As *IF* values are controlled by the interactions between metals and organics, both concentrations and compositions of metal and organics are expected to influence the extent of these interactions. In this section, we will review the findings that support this hypothesis.

### 4.1 Concentrations of Metals and Organics

Most studies have explored interaction effects using fixed metal and organic concentrations. However, several investigations demonstrate that *IF* values can vary with concentration, even within the same metal−organic mixture.^19,21,36,37,41,45^ These studies typically employed fixed concentrations for one component while varying the other. As summarized in Figure 5, although available data are limited, *IF* values can increase by as much as 1 order of magnitude with increasing organic-to-metal ratios.

Notably, Guo et al. systematically explored binary mixtures of metals and quinones over a broad concentration range (0−5 μM Fe/Cu/Mn, 0−0.1 μM PQ, 0−0.5 μM 1,2-NQ, and 0−0.5 μM 1,4-NQ).^19^ Three-dimensional plots of OP versus concentration revealed complex, concentration-dependent *IF* behavior. For example, they observed that Mn exhibited both antagonistic and synergistic interactions with PQ (*IF* = 0.53−1.49) and 1,2-NQ (*IF* = 0.51−1.63), depending on Mn concentration. More specifically, synergistic effects were observed only at relatively high Mn concentrations (>2 μM), while suppression dominated at lower concentrations. Therefore, to better understand the concentration-dependent interaction effects, future studies should prioritize matrix-based experimental designs that systematically vary metal and organic concentrations within physiologically relevant ranges.

**Figure 5:**
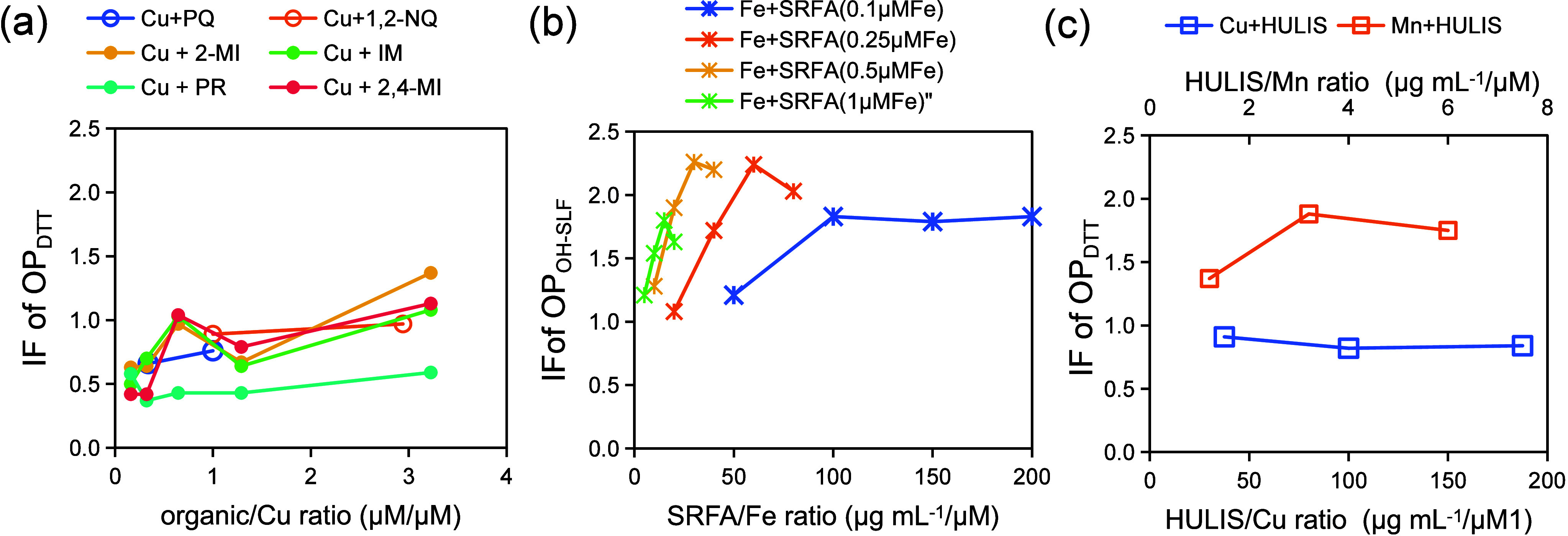
IF values in different metal−organic mixtures (IFs of OP_DTT_ for Cu-quinone were obtained from Table 2, Figures 2 and 3in Pietrogrande et al.;^41^ IFs of OP_DTT_ for Cu-imidazoles were extracted from Figure 2 in Wu et al.^45^ using Digitizer toolbox in Origin; IFs of OP_OH-SLF_ for Fe-SRFA were calculated using data from Figure 3 and Figures S6 and S7 in Gonzalez et al.^36^ using the Digitizer toolbox in Origin; IFs of OP_DTT_ for Cu/Mn-HULIS were obtained from our previous work^37^.

To inform the selection of relevant concentration ranges in acellular assays, we reviewed the ambient abundances of Fe, Cu, Mn, and HULIS and estimated their concentrations in human lung fluid from inhalation of PM for 1 day (Figure 6). Assuming a lung fluid volume of 25 mL and an inhaled air volume of 20 m^3^ per day,^68^ and applying water solubility factors of 6% for Fe, 43% for Cu, and 46% for Mn (Figure 6a), the median concentrations in lung fluid at industrial sites were 0.74 μM for Fe, 0.10 μM for Cu, and 0.30 μM for Mn. These concentrations were higher than those estimated for urban sites (0.16 μM Fe, 0.06 μM Cu, 0.11 μM Mn) and rural sites (0.13 μM Fe, 0.02 μM Cu, 0.06 μM Mn). It is evident that Fe concentrations are substantially greater than those of Cu and Mn, and that metal concentrations vary markedly by location. For HULIS, estimated concentrations ranged from 0.54 to 6.6 μg/mL, with a median value of 1.96 μg/mL.

**Figure 6:**
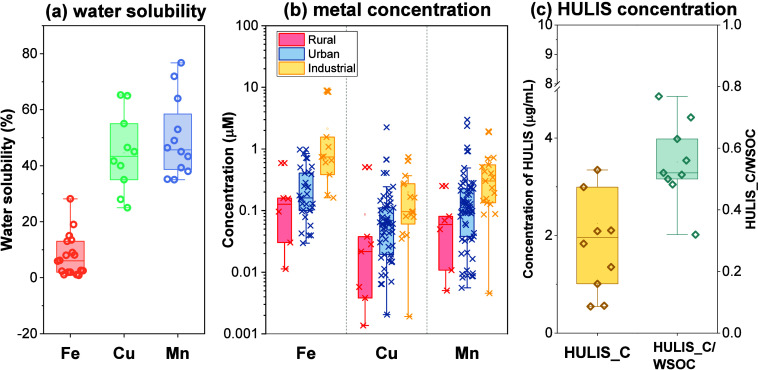
Compilation of published PM chemical characteristics relevant to oxidative potential: (a) water solubility of metals, (b) estimated metal concentrations in human lung fluid, and (c) estimated concentrations of HULIS in human lung fluid and the HULIS carbon-to-WSOC ratio (HULIS_C/WSOC). Data are drawn from studies published since 2015.^69–96^

### 4.2 Compositional Complexity in Real Samples

Solid-phase extraction (SPE) can be used to separate bulk water-extracted PM into two fractions based on hydrophilicity: HULIS and a hydrophilic fraction. Several studies have investigated the interaction effects between these two fractions in real-world PM_2.5_ samples.^21,38,39^ Using SPE cartridges packed with hydrophilic N-vinylpyrrolidone and lipophilic divinylbenzene sorbents, strongly hydrophilic substances and low molecular weight organic acids (e.g., oxalic acid) are eluted in the hydrophilic fraction with minimal retention. This effluent fraction contains most of the water-soluble metals,^12^ inorganic ions (e.g., sulfate, nitrate, ammonium), and low molecular weight organic acids (e.g., oxalic acid, acetic acid, succinic acid, malic acid, etc.).^97^ In contrast, ultrahigh-resolution mass spectrometry (UHRMS) has revealed the presence of quinone-like species, organics with carboxylic ligands, and reduced nitrogen groups (e.g., imidazole and pyridine moieties) within HULIS.^18,98,99^ These distinct organic components can form complexes with TMs, leading to variable *IFs* as discussed earlier. Consequently, this compositional complexity means that both synergistic and antagonistic interactions can coexist within these mixtures, and the overall impact on OP could be synergistic, additive, or antagonistic. For example, the interaction effects on OP_DTT_ in mixtures of HULIS and the hydrophilic fraction have been reported as both antagonistic (*IF* = 0.80 ± 0.08 ^21^) and synergistic (*IF* = 0.99−1.26 ^39^).

To explain the discrepancy, we compared the methodologies used in two studies by Yu et al.^21^ and Lin et al.^39^ Three key differences were identified: (1) the HULIS extraction protocol (ultrasonic bath^21^ vs vortex mixing^39^), (2) the presence^21^ or absence^39^ of acidification during water extraction, and (3) the distinctly different sampling locations. Previous research has shown that extraction methods (rotary agitator, ultrasonic bath and vortex) yielded no significant differences for OP_DTT_.^100^ By contrast, acidification during water extraction can substantially reduce residual metals in HULIS, as noted by Yu et al.^21^ In contrast, Lin et al.^39^ did not report employing this acidification process. Without acidification, more residual metals may remain associated with HULIS, reducing available binding sites in HULIS and altering apparent *IFs* through interactions between residual metals and the hydrophilic fractions, which itself varies in composition. Notably, Lin et al.^39^ attributed their observed discrepancy in *IFs* to compositional differences in the hydrophilic fraction. Specifically, they reported that atmospheric PM in Xi’an, China is enriched in water-soluble Fe and that synergistic ROS production between Fe and HULIS likely played a dominant role in the interaction effects. However, a more definitive comparison is constrained by a lack of reported TM and HULIS concentrations and compositions in these studies. Future investigations of PM_2.5_ component interaction effects on OP would benefit from larger ambient data sets paired with detailed chemical composition characterization.

Seasonal variability in PM_2_._5_ interaction effects is also anticipated, as inferred from different binding capacities of HULIS/WSOC with TMs across seasons.^52,101^ Seasonal shifts in HULIS chemical composition, for example, higher aromaticity in winter and higher carboxylate content in summer, may alter their complexation with TMs and thereby modulating OP interaction effects. Sources of HULIS also strongly influence its chemical composition and, consequently, its interaction with TMs and effect on OP. In our previous work,^20^ ambient HULIS had negligible effects on OP_AA_ and OP_OH-AA_ by Cu. However, *IF* values differed substantially for Cu mixtures with HULIS derived from different biomass sources: for rice straw burning HULIS, *IF* values for OP_AA_ and OP_OH-AA_ were 0.80 and 0.69, respectively, whereas for sugar cane leaf burning HULIS, the *IF* values were 1.35 and 2.71. UHRMS analysis revealed a much higher proportion of CHN species in rice straw burning HULIS (21%) compared to sugar cane leaves (1%), while both had similar levels of CHO species (62% vs 61%). This suggests that the interaction effects involving rice straw burning HULIS may be more influenced by alkaloids, whereas carboxylate-like organics likely dominate in sugar cane leaf burning HULIS.

### 4.3 Interference from Trace Metals

The nonlinear concentration−response relationship observed for Cu and Mn^11^ means that adding these metals can lead to apparent antagonistic effects, potentially confounding the assessment of true interactions in Cu− or Mn−HULIS mixtures. It is clear that although the acidification prior to the HULIS isolation makes the HULIS fractions largely metal-free, some residue amounts can remain. Previous studies have shown that up to 10% for Fe, 17% for Cu and 25% for Cr may be retained in the HULIS fraction.^12^ The concentrations of residual metals associated with HULIS can reach 0.06−0.11 μM for Cu and 0.008−0.031 μM for Mn in the DTT assay.^37^

Given that the concentrations of Cu and Mn in reaction solutions are typically as low as around 0.05−5 μM and 0.5−5 μM, respectively, in the reviewed studies, these residual levels could represent a nonnegligible proportion for Cu-HULIS mixtures in the DTT assay. In our previous work,^37^ we observed that the *IF* value for a mixture containing 0.5 μM Cu and 8 mg/L HULIS increased from 0.84 ± 0.08 to 1.00 ± 0.10 after treating HULIS samples with Chelex 100 resin to remove trace metals. Thus, residual trace metals in HULIS may interfere with the evaluation of interaction effects and should be carefully considered in experimental design and interpretation.

## 5 Implications and Future Perspectives

Based on current literature, there is growing recognition of the importance and complexity of mixture effects between TMs and atmospheric organics in determining measured OP. Generally, three key implications can be drawn from the preceding review. First, while this study has yielded conclusions regarding the impacts of different metals and organic species on *IFs* for well-investigated OP metrics (OP_DTT_ and OP_AA_), the applicability of these conclusions to other OP systems remains uncertain and requires further exploration. Second, even for OP_DTT_ and OP_AA_, critical knowledge gaps still exist, i.e., a comprehensive mechanistic framework is still lacking even for well-studied systems (e.g., metal−quinone mixtures), and investigations into real ambient PM samples remain insufficient. Although we can confirm that interaction effects exist and are influenced by metal concentrations and organic compositions, the specific mechanisms linking changes in these concentrations/compositions to variations in interaction effects remain unclear. Thus, more studies using a broader range of real samples across seasons and geographic locations, coupled with detailed compositional analyses, are necessary. Third, given the demonstrated significance of interaction effects on OP, it can be inferred that source apportionment efforts for OP should account for the interaction effects between species emitted from different pollution sources.

Substantial research gaps thus exist and require further investigation. To advance our understanding of these mechanisms and to prevent misinterpretation of OP measurements in source apportionment studies, several research directions should be prioritized.

First, a more comprehensive characterization of interaction effects is needed. Given the chemical complexity of PM, future studies should move beyond testing arbitrary metal−organic combinations, but rather employ matrix-based designs to systematically map *IF* response surfaces across concentration gradients of key species. For example, systematic investigations of •OH formation using various combinations and concentrations of Fe, Cu, Mn, HULIS, and quinones would provide valuable empirical data. Such work would clarify how mixture effects depend on component concentrations and improve the assessment of OP based on key contributors. Furthermore, because the particle size distribution of metals and organics within PM may lead to mixture effects that vary across different size fractions, and since particle size influences deposition in the respiratory tract, investigating size-segregated mixture effects is warranted. The impact of HULIS on OP is also closely linked to its chemical composition, which can vary by source. For instance, HULIS derived from biomass burning may interact differently with TMs compared to those from coal combustion. Integrating studies of TM−HULIS mixtures with molecular-level characterization, such as the relative abundance of organic classes like CHO and CHN, may yield deeper insights into the specific species responsible for these interactions.

Second, mechanistic elucidation remains a key research priority. Kinetic modeling has advanced our understanding of OP mechanisms in mixtures, particularly regarding OP_AA_ and OP_OH_.^33,36,102^ Extending these modeling efforts to other OP systems, such as OP_DTT_, will help clarify the mechanistic pathways leading to various OP end points. As OP is intimately tied to the redox properties of constituent species (e.g., TMs, quinones), electrochemical techniques, such as cyclic voltammetry, can provide direct insights into the redox properties of constituent species and their interactions. Such studies should encompass not only single organic compounds and their mixtures, but also complex atmospheric organic matrices like HULIS, to better represent ambient PM composition.

Third, improving the prediction of OP from PM composition is critical for more reliable health risk assessment. Conventional linear regression assumes additivity among components and therefore inadequate when OP is governed by nonadditive interactions. Recent advances include incorporating *IF* values,^42^ regression equations based on experimental data,^34^ interaction-based OP models,^19^ and machine learning approaches (ML).^103^ ML is particularly well suited to this problem because it can capture nonlinear relationships, uncover patterns in high-dimensional compositional data, and integrate multiple interaction features. However, all of these approaches require broader validation across diverse PM sources and environmental conditions to ensure robustness and generalizability. In particular, ML models demand large, high-quality training and validation data sets, ideally paired with standardized OP protocols and detailed chemical characterization.

Last but not least, improved health relevance is essential for translating mechanistic insights into practical benefits. Interaction effects between PM_2.5_ components were reported to influence health outcomes,^104^ suggesting that mechanistic insights gained through acellular assays could inform disease pathways. However, both positive and null associations were observed between acellular OP and biological effects in experimental studies in vitro (e.g., cellular ROS, DNA damage, cytotoxicity) and in epidemiological studies (e.g., pulmonary diseases and cardiovascular health).^9,31^ This inconsistency partly reflects the chemical and operational nature of acelular OP assays, which limits their direct relevance to cellular OP and biological outcomes.^22,31^ Specifically, most acellular OP measurements are conducted under highly artificial settings in simplified buffer systems that differ markedly from physiological extracellular fluids, in terms of ionic strength, ligands, and antioxidant capacity. As a result, interaction effects observed in acellular OP assays may not reliably correspond to those that occur in cellular assays or in vivo, where robust antioxidant buffering systems modulate redox chemistry.^31^ Furthermore, acellular assays implicitly treat all compounds as equally accessible, overlooking the cell membrane as a selective barrier. In other words, TMs and organics associated with particles of different size, polarity, and surface chemistry can differ in their ability to cross cell membranes.^105,106^ This difference in membrane penetration potential, in turn, alters the nature of TM-organic interaction effects. Moreover, acellular OP focuses on oxidative stress, whereas PM-induced biological effects involves multiple pathways beyond redox reactions. For example, polycyclic aromatic hydrocarbons and dioxin-like compounds activate the aryl hydrocarbon receptor, triggering downstream transcriptional responses.^107^ Bridging these gaps will require integrated study designs that pair acellular metrics with cellular and in vivo models under physiologically relevant conditions, and that incorporate detailed chemistry and bioavailability-relevant exposure metrics, to directly quantify net mixture effects on toxicity.

In summary, elucidating metal−organic interactions in atmospheric PM is key to advancing mechanistic understanding and improving the health relevance of OP-based assessments. Addressing these needs, through integrated assays, comprehensive chemical characterization, and bioavailability-informed exposure metrics, will enable more accurate predictive models and support more effective regulatory strategies.
